# Potential Therapeutic Use of the Rosemary Diterpene Carnosic Acid for Alzheimer’s Disease, Parkinson’s Disease, and Long-COVID through NRF2 Activation to Counteract the NLRP3 Inflammasome

**DOI:** 10.3390/antiox11010124

**Published:** 2022-01-06

**Authors:** Takumi Satoh, Dorit Trudler, Chang-Ki Oh, Stuart A. Lipton

**Affiliations:** 1 Department of Anti-Aging Food Research, School of Bioscience and Biotechnology, Tokyo University of Technology, 1404-1 Katakura, Hachioji 192-0982, Japan; 2 Departments of Molecular Medicine and Neuroscience and Neurodegeneration New Medicines Center, The Scripps Research Institute, La Jolla, CA 92037, USA; dtrudler@scripps.edu (D.T.); changki@scripps.edu (C.-K.O.); 3 Department of Neurosciences, University of California San Diego School of Medicine, La Jolla, CA 92093, USA

**Keywords:** Alzheimer’s disease, Parkinson’s disease, long-COVID, microglia, carnosic acid, rosemary, NLRP3 inflammasome, NRF2 activators

## Abstract

Rosemary (*Rosmarinus officinalis* [family Lamiaceae]), an herb of economic and gustatory repute, is employed in traditional medicines in many countries. Rosemary contains carnosic acid (CA) and carnosol (CS), abietane-type phenolic diterpenes, which account for most of its biological and pharmacological actions, although claims have also been made for contributions of another constituent, rosmarinic acid. This review focuses on the potential applications of CA and CS for Alzheimer’s disease (AD), Parkinson’s disease (PD), and coronavirus disease 2019 (COVID-19), in part via inhibition of the NLRP3 inflammasome. CA exerts antioxidant, anti-inflammatory, and neuroprotective effects via phase 2 enzyme induction initiated by activation of the KEAP1/NRF2 transcriptional pathway, which in turn attenuates NLRP3 activation. In addition, we propose that CA-related compounds may serve as therapeutics against the brain-related after-effects of SARS-CoV-2 infection, termed “long-COVID.” One factor that contributes to COVID-19 is cytokine storm emanating from macrophages as a result of unregulated inflammation in and around lung epithelial and endovascular cells. Additionally, neurological aftereffects such as anxiety and “brain fog” are becoming a major issue for both the pandemic and post-pandemic period. Many reports hold that unregulated NLRP3 inflammasome activation may potentially contribute to the severity of COVID-19 and its aftermath. It is therefore possible that suppression of NLRP3 inflammasome activity may prove efficacious against both acute lung disease and chronic neurological after-effects. Because CA has been shown to not only act systemically but also to penetrate the blood–brain barrier and reach the brain parenchyma to exert neuroprotective effects, we discuss the evidence that CA or rosemary extracts containing CA may represent an effective countermeasure against both acute and chronic pathological events initiated by SARS-CoV-2 infection as well as other chronic neurodegenerative diseases including AD and PD.

## 1. Introduction

### 1.1. Anti-Inflammatory Actions of Rosemary and Carnosic Acid

In this study, we present both an overview and new primary data describing the therapeutic effects of the herb rosemary, concentrating on its primary medicinal constituent, carnosic acid (CA). We focused on the effects of CA on neurological disorders including Alzheimer’s disease (AD), Parkinson’s disease (PD), and infection with SARS-CoV-2 causing coronavirus disease 2019 (COVID-19), as well as the long-term consequences of the infection, termed long-COVID. The anti-inflammatory action of CA through inhibition of the nucleotide-binding oligomerization domain-like receptor containing pyrin domain 3 (NLRP3) inflammasome is highlighted as a central pathway in promoting health by rosemary/CA. The keywords used as search terms for our literature review were ‘*Rosmarinus officinalis*’, ‘rosemary’, ‘carnosic acid’, ‘carnosol’, ‘NLRP3′, ‘Alzheimer’s disease’, ‘Parkinson’s disease’, ‘COVID’, and ‘NRF2′. All types of articles, abstracts, and books were included in the search. No time limitation on publication date was considered.

### 1.2. Uses of Rosemary and Rosemary Extract

Rosemary is an economically important plant species that is widely distributed due to its culinary, medicinal, and cosmetic uses [1,2,3]. Both fresh and dried leaves of rosemary have been used for their characteristic aroma and flavor in many food dishes. Additionally, rosemary extracts have been employed as lipophilic antioxidants to preserve foods against oxidation from environmental stress [1,2,3]. Because of these characteristics, the European Union, United States, and Japan have all approved rosemary extracts as food additives for food preservation [4,5,6]. Rosemary contains two groups of potentially active compounds. One group is comprised of small molecular weight aromatic compounds, called “essential oils”, which rapidly evaporate to produce the characteristic smell and taste of rosemary. The other group is represented by the polyphenolic compounds including carnosic acid (CA) and carnosol (CS), which have been shown to manifest direct and indirect antioxidant actions [7,8,9]. While our group has focused on the effects of CA, we also report here the work of others on CS.

## 2. Anti-Inflammatory Mechanisms

### 2.1. Anti-Inflammatory Effects of CA on Tissue Macrophages

**Figure 1:**
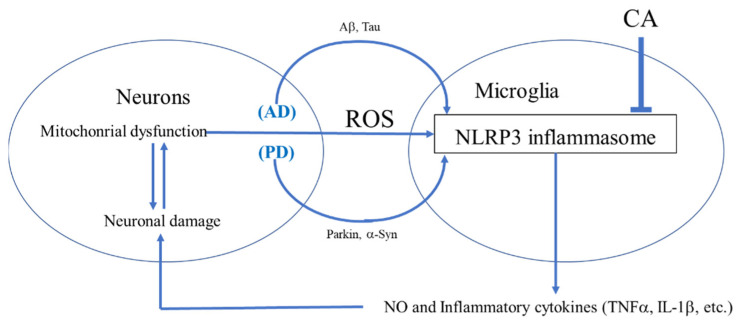
**Microglial-mediated inflammation in neurodegenerative disorders.** During neurodegenerative disorders such as AD and PD, various misfolded proteins from neurons or other cell types trigger microglial inflammation [18,19,20,21,22,23,24,25,26,27]. In response to these stimuli, activated microglia release NO and inflammatory cytokines. CA may block the inflammatory loop between neurons and microglia through inhibition of the NLRP3 inflammasome [28,29,30].

While CA may act on a variety of cell types, we concentrated here on monocytoid cells of the innate immune system. CA-mediated modulation of the macrophage and microglial inflammatory response in AD and PD via inhibition of NLRP3 could potentially have significant therapeutic value (Figure 1) [10,11]. Along these lines, studies have revealed that rosemary extract or CA can inhibit inflammatory cytokine expression (i.e., tumor necrosis factor-*α* (TNF-*α*)), in Raw 264.7 macrophage cells [10,11]. CA also attenuates LPS-induced nitric oxide (NO)/reactive nitrogen species (RNS) production in these macrophage cells [11]. Furthermore, CA can suppress the TNF-*α* signaling pathway by inhibiting the inhibitor of nuclear factor κ-B (NF-κB) as well as via upregulation of HO-1 expression [12,13]. As discussed in more detail below, by upregulating erythroid derived 2-related factor 2 (NRF2) transcriptional activity, CA leads to the downregulation of the TNF-*α* and NO inflammatory response [14,15,16,17]. Moreover, in vivo, CA has also been shown to inhibit the LPS-induced rise in serum levels of proinflammatory cytokines including TNF-*α* and interleukin (IL)-6; many of these effects of CA are due to NRF2 activation [14]. For these in vivo studies, CA was suspended in 0.5% solution and administered (100 mg/kg) by gavage once daily for three days. Activation of NRF2 was confirmed by NRF2 nuclear translocation and increased expression of antioxidant genes known to be transcriptionally regulated by the NRF2 pathway [14].

### 2.2. Anti-Inflammatory Effects of CA on Brain Microglia

**Figure 2:**
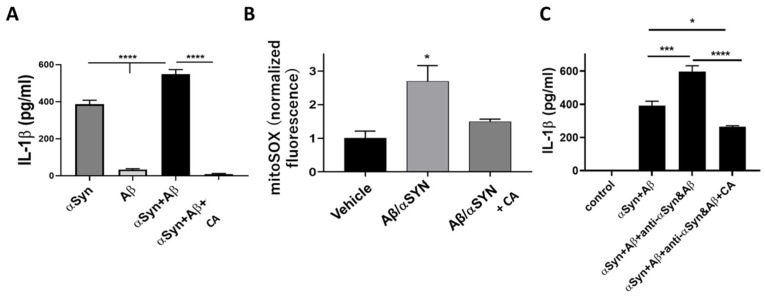
**Carnosic acid (CA) prevents NLRP3 inflammasome activation in human iPSC-derived microglia (hiMG).** hiMG were exposed to CA (2 µM) for 16 h and then to misfolded Aβ (500 nM oligomers) plus a low concentration of αSyn (150 nM oligomers) for 6 h. (**A**) Under these conditions, CA (2 µM) completely abrogated inflammasome activation, evidenced by decreased IL-1β release measured by ELISA, as described previously [28]. (**B**) CA attenuated hiMG mitochondrial ROS production, which provides Signal 2 (in Figure 3) for NLRP3 inflammasome activation. Mitochondrial ROS was monitored with the fluorescent probe mitoSOX, as described previously [28]. (**C**) CA (2 µM) significantly ameliorated IL-1β release from hiMG exposed to oligomeric Aβ plus low concentrations of oligomeric α-Syn in the presence of their respective human antibodies (anti-αSyn or anti-Aβ). Values are mean + S.E.M., *n* = 3 per group, * *p* < 0.05, *** *p* < 0.001, **** *p* < 0.0001 by ANOVA with Bonferroni post hoc test.

Microglial cells are the major resident innate immune inflammatory cells of the brain and, upon activation, can produce proinflammatory cytokines (e.g., IL-1β, IL-6, and TNF-*α*). In an in vitro model of brain infection, CA was found to inhibit LPS-induced activation of mouse microglia, thus decreasing the release of inflammatory cytokines such as IL-1β and IL-6 [15,16,17]. CA is also reported to decrease NO production associated with inducible NO synthase [15,16,17]. Several studies have observed that misfolded proteins such as α-synuclein (α-Syn), particularly in combination with amyloid-β (Aβ) or possibly Tau and release of inflammatory cytokines from microglia is critical for microglial–astrocyte–neuron signaling in neurodegenerative disorders (Figure 2) [18,19,20]. In addition, microglial-mediated inflammation is reportedly upregulated in human AD and PD brains [18,19,20]. Based on various inflammatory models, the therapeutic potential of CA has been suggested for a number of neurodegenerative disorders including AD and PD [18,19,20].

## 3. Role of NLRP3 Inflammasome

### 3.1. Proteins of the NLRP3 Inflammasome

**Figure 3:**
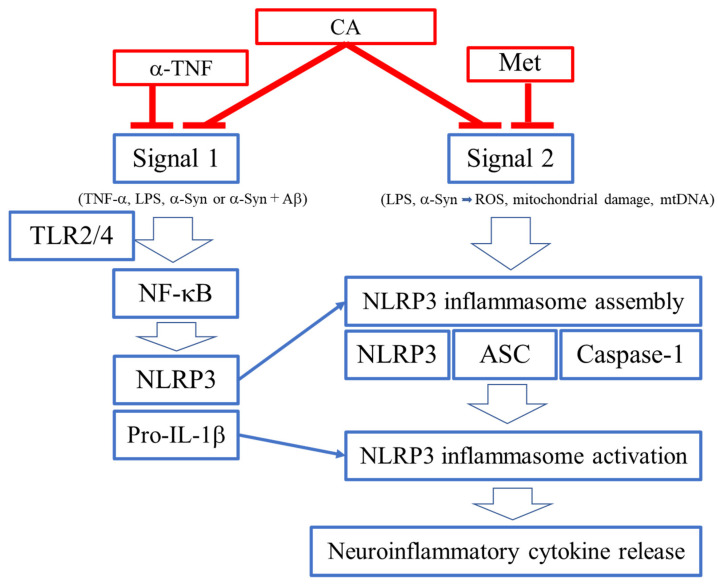
**Activation of NLRP3 inflammasome.** AD, PD, and many other neurodegenerative disorders can be triggered by the accumulation of misfolded proteins such as Aβ, Tau, Parkin, and α-Syn [18,19,20]. Two signals are involved in NLRP3 activation during inflammation that accompanies and contributes to AD and PD pathogenesis [28,29,30,31]: Priming and activation. For Signal 1, priming, an extracellular signal such as TNF-α, LPS, or α-Syn activates NF-κB, generally via Toll-like receptors, culminating in formation of an inflammasome complex with NLRP3, caspase-1, and ASC. Interestingly, in PD and LBD, misfolded α-Syn can trigger the NLRP3 inflammasome by providing both priming and activation (Signal 1 and Signal 2, respectively) [32,33].

Caspases are cysteine proteases that play essential roles in apoptosis and other cellular events [21,22]. There are two types of caspases in terms of their functions during apoptotic pathways, initiators such as caspase-1 and executors such as caspase-3 [21,22]. Caspase-1 is an initiator of inflammation as well as apoptosis (Signal 1 and Signal 2 in Figure 3) [21,22]. Caspase 1 can be activated by the formation of molecular complexes via protein–protein interactions [23,24,25]. Caspase-1 contains a prodomain and forms a large protein complex [23,24,25]. The multimeric protein complexes are termed “inflammasomes” [23,24,25]. The complexes include a sensor protein, termed NLRP3, an adaptor protein, ASC (apoptosis-associated speck-like protein containing a CARD), and pro-caspase-1 [23,24,25]. The sensor binds various stress factors such as exogenous pathogens and endogenous molecules, and subsequently induces oligomerization of ASC in the large complex [23,24,25]. Upon pro-caspase-1 binding, the protease is activated by an autocatalytic process and in turn activates pro-caspase-1 to active caspase-1, which then cleaves the proinflammatory cytokines pro-IL-1β and pro-IL-18 to their active forms [23,24,25]. Secretion of the mature/active forms of these cytokines (IL-1β and IL-18) induces inflammation [21,22]. Additionally, active caspase-1 can cleave gasdermin D, whose N-terminal fragment translocates to the outer membrane where it forms large pores, resulting in a lytic form of cell death (pyroptosis) [21,22].

### 3.2. Signals 1 and 2 for Activation of NLRP3 Inflammasome

Although molecular details involved in activation of the NLRP3 inflammasome remain to be elucidated, two signals are known to be involved (Signals 1 and 2 in Figure 3) [26,27]. TNF-α stimulates its cognate receptor or other initiators such as LPS stimulate Toll-like receptors (e.g., TLR4) on immune cells as “Signal 1.” Misfolded α-synuclein (α-Syn) protein can provide Signal 1 via interaction with TLR2, and can also provide Signal 2, possibly by triggering production of reactive oxygen species (ROS) [28]. Signal 1 involves the expression of NLRP3, pro-IL-1β, and pro-IL-18, mediated through NF-κB activation. Signal 2 is activated by various types of stress such as ROS, mitochondrial damage, mitochondrial (mt)DNA, or misfolded proteins such as α-Syn, particularly in combination with amyloid-β (Aβ) (Figure 2) or possibly other misfolded proteins like Tau [28,29,30,31]. Several groups have shown that ROS can play a major role as a mediator of Signal 2 for NLRP3 inflammasome activation [32,33]. Because NRF2, as discussed further below, is a master regulator of cellular defense against oxidative stress, NRF2 activators would be expected to downregulate ROS and hence limit NLRP3 inflammasome activation [34,35,36,37,38,39,40].

### 3.3. Activation of NLRP3 Inflammasome by Misfolded Proteins

Recently, the accumulation of various misfolded proteins has been reported to induce activation of neuroinflammatory pathway signaling between microglia and neurons in AD, PD, and other neurodegenerative disorders. NLRP3 inflammasome activation in microglia represents one such mediator [21,22,23,24,25,26,27]. For example, we recently reported that misfolded α-Syn, akin to LPS, can induce activation of NLRP3 inflammasome in human induced pluripotent stem cell (hiPSC)-derived microglia (providing both Signal 1 and Signal 2 in Figure 3); Aβ oligomers (prepared and analyzed as described in [28] and references therein) enhance this signaling in hiPSC-derived microglia [28]. Furthermore, this work showed that antibodies directed against misfolded proteins such as α-Syn and Aβ actually increase NLRP3 activation, contributing to neuroinflammation [28]; despite this, these antibodies are being used in human clinical trials for AD and PD, and, in a contentious decision, one such antibody therapy was recently approved by the FDA for the treatment of mild AD.

## 4. Inhibition of NLRP3 Activation by CA

### 4.1. Activation of the NLRP3 Inflammasome Complex

Although various molecules can activate the NLRP3 inflammasome, ROS, for example, originating from mitochondrial dysfunction, may play a central role in this activation [40,41]. Because accumulation of misfolded proteins in neurons and released from neurons induces mitochondrial dysfunction and ROS/RNS production, these pathological events may contribute to neuroinflammatory signaling between neurons and microglia [28,29,30,31]. At least two molecular mechanisms have been proposed that lead to NLRP3 activation in response to ROS [42,43,44]. One possibility involves thioredoxin-interacting protein (TXNIP), which binds to thioredoxin under normal conditions, but is released from the complex by oxidative stress and then binds to NLRP3 to activate the inflammasome [42]. Another proposed mechanism involves mitochondrial antiviral signaling protein (MAV) [43,44]. MAV is normally localized in mitochondria, but during inflammation, it diffuses in response to mitochondrial dysfunction into the cytoplasm to bind to the NLRP3 inflammasome and activate the complex [43,44]. As the molecular details for activating the NLRP3 inflammasome are still emerging, additional signals may also come to light [42,43,44].

### 4.2. ROS as a Signal 2 Activator and Therapeutic Target

CA has been proposed as a therapy to inhibit oxidative stress that occurs with severe inflammatory conditions [32,33,34,35]. Because the second signal for NLRP3 inflammasome activation can be ROS (Signal 2 in Figure 3) [32,33], the anti-inflammatory actions of CA may be related to its antioxidant properties [36,37,38,39,40]. Along these lines, reports using various disease models suggest that NRF2 activators can regulate the NLRP3 inflammasome through the reduction in oxidative stress [45,46,47]. In addition, CA can attenuate the NF-κB signaling pathway that contributes to NLRP3 activation (Signal 1 in Figure 3) [45,46,47]. Collectively, the anti-inflammatory effects of NRF2 activators may manifest in part by the inhibition of the NLRP3 inflammasome (via Signal 1 and Signal 2 in Figure 3) [45,46,47].

### 4.3. In Addition to α-Syn and Aβ, Possibly Other Misfolded Proteins May Contribute to Activation of the NLRP3 Inflammasome through ROS Generation

Interesting, recent evidence suggests that while one or a few misfolded proteins may predominate in a given disease (e.g., Aβ and Tau in AD), other misfolded proteins that are usually associated with another disease (e.g., α-Syn in PD and Lewy body dementia [LBD]) can also be present; for example, more than half of human AD brains have been found to contain misfolded α-Syn, and PD/LBD brains contain Aβ and Tau in addition to predominant α-Syn (for references, see [28]). These and other misfolded proteins can induce mitochondrial dysfunction in neurons and, upon their release, in surrounding neurons, astrocytes, and microglia. One consequence of these misfolded proteins is the overproduction of ROS, contributing to the activation of the NLRP3 inflammasome, and release of proinflammatory cytokines from microglia [28,29,30,31]. These cytokines can then contribute to synaptic and neuronal damage. Thus, the NLRP3 inflammasome has emerged as a plausible therapeutic target for AD and PD [28,29,30,31].

### 4.4. CA as an Inhibitor of NLRP3 Inflammasome Activation in PD- and AD-Related Models

Collectively, Signal 1 is an initiator and Signal 2 is an activator of the NLRP3 inflammasome. CA reportedly inhibits LPS-induced inflammation of microglia, implying inhibition of the inflammasome. Moreover, the Karin group in collaboration with two of us (DT, SAL) reported that metformin (Met) significantly decreased Signal 2 of the NLRP3 inflammasome [48]. In contrast, anti-TNF-α antibody (α-TNF) suppresses Signal 1 in Figure 3 [49]. Many reports have demonstrated the anti-inflammatory effects of CA in various model systems dependent on TNF-α- and LPS-mediated activation of the NLRP3 inflammasome, thus limiting the release of proinflammatory cytokines [45,46,47]. With this background, we considered the possibility that CA could inhibit NLRP3 activation in the context of neurodegeneration. However, there has been no direct evidence for CA-mediated inhibition of the NLPR3 inflammasome activity triggered by misfolded proteins. Accordingly, we demonstrate that IL-1β release due to NLRP3 inflammasome activation by misfolded α-Syn ± Aβ [28] is inhibited by CA in hiPSC-derived microglial cells (Figure 2). Moreover, the enhanced activation of the NLRP3 inflammasome, and thus increased IL-1β release, observed in the added presence of anti-α-Syn or anti-Aβ antibodies [28] was also abrogated by CA (Figure 2), implying that this type of therapeutic intervention might offset at least some of the inflammatory side effects of these antibody therapies being developed for AD and PD.

## 5. Direct and Indirect Antioxidant Actions of Rosemary and CA

### 5.1. Direct Antioxidant Action

**Figure 4:**
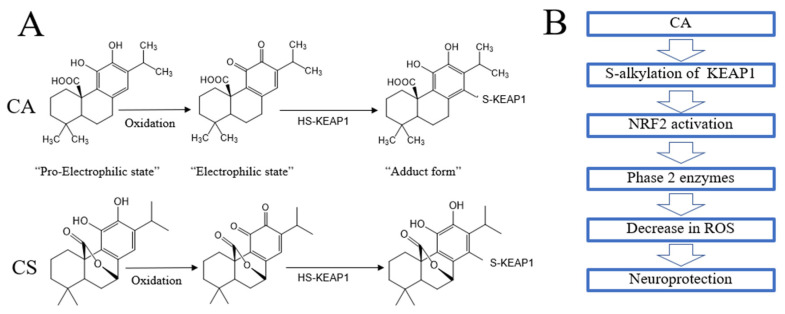
**Pro-electrophilic drugs (PEDs) acting as a pathologically-activated therapeutic (PAT) via NRF2 stimulation.** (**A**) PAT drugs [37,38]. Carnosic acid (CA) from *Rosmarinus officinalis* is a catechol-type pro-electrophilic drug (PED) [36,37,38]. Oxidative activation of the “pro-electrophilic state” to the “electrophilic state” requires electron acceptors such as ROS [51,52,53,54,55]. These compounds then react with cysteine thiols after they are oxidized to their electrophilic/quinone state [36,37,38]. For this reaction, a cysteine thiol triggers nucleophilic attack of the electrophilic compound to form an adduct [36,37,38]. Thus, CA can transform from a non-active (pro-electrophilic) state to an active (electrophilic) state under the oxidative stress [36,37,38]. CA is thus activated only in tissue undergoing oxidative stress and can in turn protect the tissue from such stress, as occurs in neurodegenerative disorders such as AD and PD as well as COVID-19. PEDs thus fulfill the concept of “pathologically-activated therapeutic (PAT)” drugs [37,38]. (**B**) Activation of the KEAP1/NRF2 pathway. The NRF2/KEAP1 pathway represents one of the major cellular defense systems against oxidative stress [34,35,36]. NRF2 is a transcription factor that induces phase 2 antioxidant enzymes. Under normal conditions, KEAP1 protein binds to NRF2 and functions as an adaptor protein for cullin 3 (encoded by Cul3 in humans) E3 ubiquitin ligase, which polyubiquitinates NRF2 [34,35,36]. Consequently, NRF2 is ubiquitinated and degraded by the proteasome. Hence, the transcriptional activity of NRF2 is potently inhibited under normal conditions. KEAP1 contains critical cysteine thiols that react with CA after electrophilic conversion [34,35,36]. This reaction prevents KEAP1 from inducing ubiquitination and degradation of NRF2. NRF2 thus dissociates from the cytoplasmic complex with KEAP1, enters the nucleus, and binds to AREs in the promoters of target phase 2 genes, which encode a coordinated system of antioxidant and anti-inflammatory enzymes. These proteins include enzymes that generate the major cellular antioxidant, glutathione (GSH). Thus, NRF2 activators protect various cell types including neurons via redox regulation [51,52,53,54,55]. In the brain, activation of NRF2 occurs mainly in astrocytes and microglial cells [15,16].

Antioxidant actions of CA and CS have been demonstrated via oxidation reactions and the protection of cells from oxidative cell death [50,51]. For example, oxidative insult via ROS is thought to convert the catechol form of CA to an *ortho*-quinone (Figure 4A) [51,52,53,54,55]. Through this mechanism, CA can protect neuronal cells against oxidative stress (e.g., due to hydrogen peroxide and lipid hydroperoxides) [51,52,53,54,55]. Thus, some of the antioxidant effects of polyphenolic natural products are due to catechol conversion to an *ortho*-quinone [36,37,38,39,40]. These biological effects of diterpenes and triterpenes are closely related to direct interactions with ROS [8,9].

### 5.2. Indirect Antioxidant Action

As alluded to above, CA also manifests prominent indirect (mainly transcriptional) antioxidant actions in addition to these direct antioxidant mechanisms. The induction of phase 2 antioxidant enzymes is an essential cellular defense against oxidative stress mediated by these compounds (Figure 4B) [36,37,38,39,40]. The NRF2 transcription factor binds to the antioxidant response element (ARE) on the enhancer/promoter region of DNA to induce the genes encoding phase 2 antioxidant and anti-inflammatory enzymes [34,35,36,37,38,39,40] including glutathione (GSH)-regulating enzymes such as GSH-S-transferase (GST) and γ-glutamylcysteine synthetase (γ-GCS), and also thioredoxin (TRX) reductase [34,35,36,37,38,39,40]. Mechanistically, S-alkylation of the cysteine thiol of the Kelch-like ECH-associated protein 1 (KEAP1) protein by the “electrophilic” quinone derivative of CA can activate the KEAP1/NRF2 pathway [34,35,36,37,38,39,40]. KEAP1 tightly binds with and retains NRF2 in the cytosol via cysteine thiols (specifically, Cys151, Cys273, and Cys288) on the KEAP1 protein. Subsequent S-alkylation (adduct formation) with a quinoid form of CA inhibits KEAP1 binding to NRF2, thus allowing translocation of the NRF2 into the nucleus [56,57,58]. There, NRF2 upregulates gene expression by binding to the AREs of phase 2 genes [54,55]. Through this type of mechanism, CA can exert neuroprotective effects in various models of central nervous system (CNS) disease [59,60,61,62]. For example, CA has been shown to protect neuronal HT22 cells against oxidative glutamate toxicity by activating the KEAP1/NRF2 pathway [50,51,52,53,54,55]. The free carboxylic acid and catechol hydroxyl moieties of CA have been shown to play a critical role in these effects [50,51,52,53,54,55]. Emerging evidence suggests that the major rosemary constituents (e.g., CA) protect neurons against oxidative stress by activating the KEAP/NRF2 pathway in this manner [50,51,52,53,54,55]. Phase 2 antioxidant/anti-inflammatory enzymes thus transcribed include heme oxygenase-1 (HO-1), NAD(P)H quinone dehydrogenase 1 (NQO-1), γ-GCS, and GST, all of which provide neuroprotection in part by upregulating the GSH-mediated redox system [50,51,52,53,54,55]. CA also protects from lipopolysaccharide (LPS or endotoxin, as found in bacterial infection)-induced liver damage through the induction of phase 2 antioxidant enzymes [15,16,17] and references therein. CA or CS significantly reduces LPS-induced generation of ROS and RNS (including NO) in RAW264.7 macrophages and is associated with the upregulation of HO-1 and NQO-1 [10,11] and references therein.

### 5.3. CA-Mediated Protection of the Brain in AD and PD

Because of these direct and indirect antioxidant mechanisms, the potential of rosemary extract or CA for the treatment of AD and PD has been discussed based on positive findings in various model systems [61,62,63,64,65,66,67,68]. Mechanistically, many studies have demonstrated that mutated or misfolded proteins associated with these neurodegenerative disorders including Aβ, Tau, TDP-43, Parkin, and α-Syn increase ROS/RNS production through mitochondrial dysfunction and contribute to synaptic damage and neuronal cell death [69,70,71,72]. Excessive stimulation of *N*-methyl-d-aspartate (NMDA)-type glutamate receptors on the surface of pyramidal neurons of the cortex and hippocampus also contributes to ROS/RNS generation and subsequent synaptic injury and neuronal loss [71,72]. Many research groups including our own have reported that CA exerts a protective effect on neuronal cells through their antioxidant mechanisms in model systems of AD and PD [66,67,68]. There is strong evidence to suggest that the generation of oxidative and nitrosative stress contributes to neuronal and synaptic damage in the pathogenesis of AD and PD [66,67]. Activation of the KEAP1/NRF2 pathway increases the transcription of phase 2 antioxidant and anti-inflammatory genes. Hence, this pathway represents a promising therapeutic approach to various neurodegenerative conditions. The protective effects on primary neurons of CA have been studied after exposure to Aβ oligomers in both in vitro and in vivo models. Both neurobehavioral and histological improvement was observed. The histological results revealed that CA (10 mg/kg, administered twice weekly for three months to the brain via the transnasal route) increased synaptic and dendritic markers, and decreased amyloid plaque number, astrogliosis, and phospho-tau staining in the hippocampus [66]. Protection in vivo by CA has been examined in PD models using 6-hydroxydopamine (6-OHDA) to injure the dopaminergic neurons in the substantia nigra [67]. Rats were treated with 20 mg/kg CA for three weeks prior to 6-OHDA exposure. CA improved the locomotor activity of the 6-OHDA-exposed rats. Significant protection against lipid peroxidation and GSH reduction was also observed in 6-OHDA rats pretreated with CA [67].

## 6. Complicated Manifestations of COVID-19

### 6.1. Manifestations

**Figure 5:**
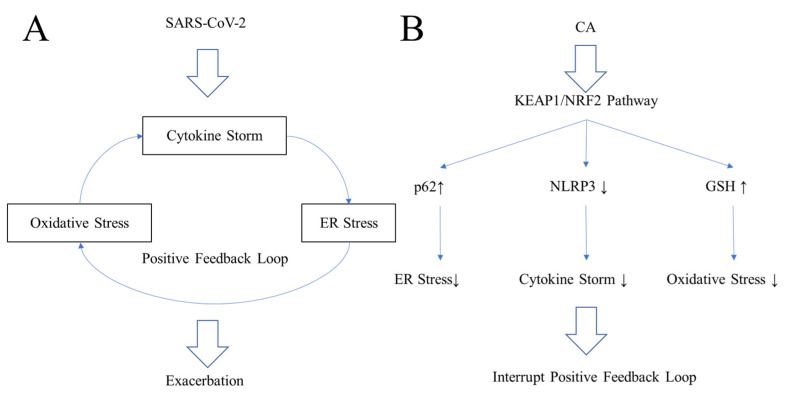
**Schema of immune-related events in COVID-19.** (**A**) Positive feedback loop of events in COVID-19. SARS-CoV-2 induces complicated manifestations due to three types of stress: Cytokine storm, oxidative stress, and ER stress, each of which stimulates the others [75,76]. (**B**) NRF2 activators to interrupt the positive feedback loop [37,38]. NRF2 activators can stimulate the KEAP1/NRF2 pathway and induce p62 and the reduced form of glutathione (GSH), important regulators of ER and oxidative stress [37,38]. NRF2 activators also suppress the NLRP3 inflammasome, a potential contributor to cytokine storm in COVID-19 [77,78].

COVID-19, caused by the virus SARS-CoV-2, has rapidly become the most urgent issue in global public health [73,74]. OVID-19 is composed of many complicated symptoms in addition to acute respiratory distress syndrome (ARDS) as well as physiological and biochemical alterations (Figure 5) [73,74]. These include the translocation of T cells into the lung, alterations in coagulation, and increased ferritin levels, a mediator of immune dysregulation [75,76]. Contributing to these manifestations is a positive feedback loop at the cellular level composed of cytokine storm, oxidative stress, and ER stress (Figure 5) [75,76]. Since few specific therapies (other than vaccination) are available, patients have been treated symptomatically with oxygen and broad-spectrum antiviral drugs such as interferon-α and glucocorticoids [75,76]. Remdesivir, a medicine used to treat Middle East Respiratory Syndrome (MERS), is at least partly effective against COVID-19 infection [75,76]. More specific therapeutic drugs, such as Pfizer’s Paxlovid and Merck’s molnupiravir, are being developed and have just been released as of this writing to supplement widespread vaccination efforts [75,76].

### 6.2. Cytokine Storm

**Figure 6:**
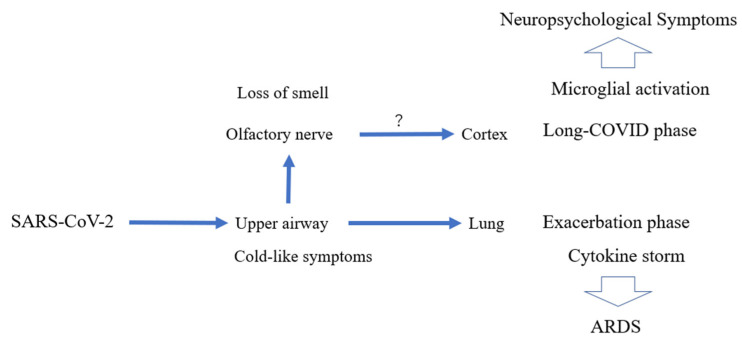
**Schema of pathological processes potentially involved after SARS-CoV-2 infection that contribute to airway and CNS symptoms.** Initially, the virus enters the body through the nose and mouth, and is trapped on the surface of the upper airway [73,74,75,76]. Although inflammation may be induced around epithelial cells, symptoms at this stage are mild with cold-like manifestations if the reactions are limited to the upper airway [73,74,75,76]. However, when the virus invades the lung epithelia, the situation worsens, leading to ARDS with cytokine storm [73,74,75,76]. Additionally, virus may affect the nervous system with loss of taste and smell [73,74,75,76]. Whether virus proliferates in sensory neurons and can be retro-transmitted to the CNS remains contentious and unproven despite some claims [73,74,75,76]. Mechanism notwithstanding, long-term symptoms of “brain fog” have been frequently reported [73,74,75,76]. These sustained after-effects have been termed “long-COVID” and may persist well into the post-infectious period [73,74,75,76]. The possibility of microglial-mediated cytokine dysfunction contributing to these symptoms, for example, affecting activity in the cingulate cortex, has been discussed [73,74,75,76].

Initially, SARS-CoV-2 infects epithelial cells of the upper respiratory tract. If the infection is limited to these cells, the disease can manifest mild symptoms (Figure 6) [77,78]. Severe inflammation develops when the virus enters the lungs [77,78]. Translocation of T lymphocytes into the alveoli may contribute to inflammation, leading to the development of ARDS [77,78]. In severe cases, the virus can enter the blood circulation and spread the infection to other parts of the body (Figure 6) [77,78]. The “damage-associated molecular patterns (DAMPs)” released from the dead cells activate immune cells and can induce cytokine storm [77,78]. Thus, in severe forms of COVID-19, hyperactivation of the immune system can contribute to multiple organ failure [77,78]. In general, tightly controlled activation of the innate immune system is essential for viral recognition and clearance of viruses [77,78]. However, dysregulated inflammation causes a cytokine storm due, in part, to NLRP3 inflammasome activation [79,80]. Cytokine storm can also contribute to hypercoagulation in microvessels, another component of multi-organ failure [79,80]. Patients in critical condition often manifest systemic inflammatory markers such as high levels of cytokines, including IL-6, TNF-α, and IL-8 [79,80]. Thus, measures to counter cytokine storm have been proposed as part of the treatment regimen [77,78,79,80].

### 6.3. Oxidative Stress in COVID-19

Inflammatory cytokines and ROS act together to activate lung epithelial cells [79,80]. This leads to dysregulated cell contact, greater cell permeability, and influx of fluid, collectively contributing to lower oxygen tension [77,78]. Oxidative stress and cytokine storm also lead to dysfunction and apoptosis of endothelial cells and activation of the coagulation system [81,82]. Accumulation of serum ferritin and Fe^3+^ have been reported in severeCOVID-19 [81,82]. Ferritin can convert Fe^3+^ to Fe^2+^, which can then participate in the Fenton reaction [81,82], contributing further to oxygen free radical production. The increase in ROS can cause additional release of iron from stores with ferritin as well as damage to DNA, lipids, and proteins [81,82,83,84]. It has been proposed that hydrogen gas or high concentrations of vitamin C can at least partially prevent these events [83,84].

### 6.4. Endoplasmic Reticulum (ER) Stress in COVID-19

SARS-CoV-2 viral proteins expressed in infected cells are folded in the host cell endoplasmic reticulum (ER) [85,86]. Viral proteins are subject to modification in the intracellular membrane of the ER and contribute to ER stress [85,86]. Replication of SARS-CoV-2 requires a large number of proteins, and after multiple replication cycles, budding leads to viral release from the host cell [87,88].

### 6.5. Inhibiting the Positive Feedback Loop of COVID-19

SARS-CoV-2 induces a positive feedback loop consisting of cytokine storm, oxidative stress, and ER stress in lung epithelia during the exacerbation phase of COVID-19 (Figure 5A) [89,90]. Due to these complex features of COVID-19, classical anti-inflammatory drugs and antioxidants cannot fully restore lung function [89,90]. One countermeasure would be to disrupt the positive feedback loop (Figure 5B) [89,90]. To effectively combat the positive feedback loop that contributes to COVID-19 pathogenesis, NRF2 activators have been proposed as a potential therapeutic [90,91]. Until now, however, most proposed therapeutic interventions have focused on overcoming each stress individually rather than the complex positive feedback loop [81,82,83,84,85,86,87,88]. For example, dexamethasone is a steroid anti-inflammatory drug, while hydrogen gas or high concentrations of vitamin C are potential free radical scavengers [83,84]. However, none of these approaches completely suppresses ARDS. A more comprehensive approach to disrupting the positive feedback loop is probably required for an effective therapy (Figure 5B) [89,90]. Prior work has shown that NRF2 activators may protect the lung and brain from severe inflammation [89,90]. NRF2 can ameliorate inflammation by reducing IL-*6* and IL-1β, in part by inhibiting activation of the NLRP3 inflammasome (Figure 5B) [89,90]. NRF2 induces the expression of genes encoding phase 2 antioxidant and anti-inflammatory enzymes, leading to an increase in GSH and preservation of redox homeostasis to protect against oxidative stress [36,37,38,39,40]. Additionally, NRF2 can induce the anchor protein, p62, which activates autophagy to ease ER stress [91,92]. Thus, NRF2 activators coordinately reduce ER stress, oxidative stress, and cytokine storm by inducing p62, inducing GSH synthetic enzymes, and inhibiting the NLRP3 inflammasome, leading to suppression of the positive feedback loop [89,90].

## 7. Potential Relationship of Neurological Sequelae in COVID-19 and Microglial Activation

Neurological symptoms induced by SARS-CoV-2 can produce cognitive symptoms called brain fog, the origin of which is still unclear. This symptom is common in what has been termed “long-COVID” [93,94]. Microglia, the innate immune cells of the CNS, are known to play a central role in the maintenance of brain homeostasis and inflammatory responses [93,94]. Normally, microglia can maintain homeostasis and contain inflammation, resulting in the improvement of symptoms [93,94]. However, during neurodegenerative disorders and possibly during COVID-19, the proinflammatory state of microglia could be deleterious. long-COVID patients persistently suffer from unacceptable neurological symptoms such as brain fog, fatigue, depression, and anxiety [93,94]. The question remains whether in long-COVID patients, microglia are hyperactivated and become proinflammatory, and if this process is accelerated by activation of the NLRP3 inflammasome [95]. If so, an inhibitor of the NLRP3 inflammasome could become an effective therapeutic for long-COVID.

## 8. CA as a Potential COVID-19 Therapeutic via Inhibition of NLRP3 Inflammasome, Other Inflammatory Pathways, and SARS-CoV-2 Infection

### 8.1. ARDS Mediated by NLRP3 Inflammasome

**Figure 7:**
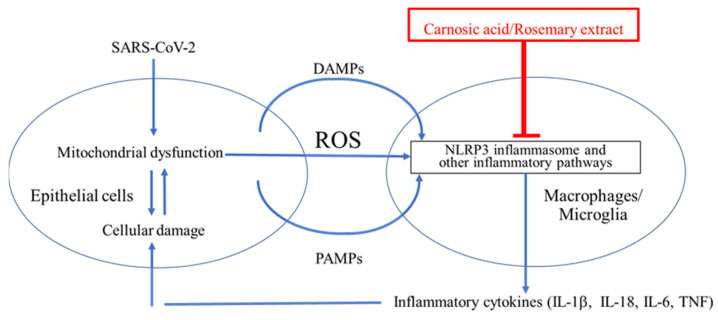
**Cytokine storm contributes to ARDS in the lungs and possibly the brain of COVID-19 patients.** SARS-CoV-2 binds to ACE2 and enters epithelial cells to induce ER stress due to overproduction of viral proteins [73,74,75,76]. The damaged cells release damage-associated molecular patterns (DAMPs), pathogen-associated molecular patterns (PAMPs), and ROS [24,25,26]. ROS, DAMPs, and PAMPs activate the NLRP3 inflammasome in surrounding macrophages in the lung and potentially in microglia in the brain [77,78]. This activation is critical for the release of inflammatory cytokines such as IL-1β from macrophages and microglia [77,78]. These cytokines enhance damage of epithelia and other cell types and may contribute to cytokine storm and the severe lung disease of ARDS [96,97]. The NLRP3 inflammasome is thus a plausible drug target for treating ARDS [77,78].

As schematically highlighted in Figure 7, ARDS is initiated by SARS-CoV-2 infection. Viral replication can lead to TLR signaling and NF-κB activation (Signal 1 in Figure 2) [96,97]. However, this signal itself is not the direct cause of ARDS [96,97]. It is the dysregulated, hyperinflammatory response to viral infection, mediated at least in part by NLRP3 inflammasome activation, which contributes to ARDS [96,97]. As reviewed above, the extreme inflammatory response is triggered by activation of the NLRP3 inflammasome and other inflammatory pathways that induce release of proinflammatory cytokines such as IL-1β, IL-18, IL-6, and TNF-α [32,33,34,35]. Thus, a potential countermeasure against the development of severe COVID-19 would be to prevent activation of NLRP3 and other inflammatory pathways [32,33,34,35].

### 8.2. CA as a Potential Treatment for COVID-19 by Suppressing Infection and Cytokine Production

Recently, McCord et al., reported that PB125, a CA-based therapeutic agent, was potentially useful in the treatment of respiratory viral diseases, including COVID-19, in part by inhibiting cytokine storm [98,99,100]. They observed a marked downregulation of genes encoding inflammatory cytokines including IL-1β, IL-6, TNF-α, cell adhesion molecules (ICAM-1, VCAM-1, and E-selectin), and a group of interferon-γ-induced genes, suggesting that cytokine storm might be suppressed by CA [98,99,100]. McCord and colleagues further reported that PB125 downregulates ACE2 and TMPRSS2 mRNA expression in human liver-derived HepG2 cells, consistent with the notion that CA might inhibit viral entry, which ACE2 and TMPRSS2 proteins mediate [98,99,100]. These results suggest that the use of rosemary extract or CA might represent a possible therapeutic against acute ARDS in the lung and more protracted cytokine production [98,99,100]. Therefore, clinical testing of rosemary extract or CA for either acute COVID-19 lung disease or chronic (so-called long) COVID-19 might be considered. The rationale for such treatment is discussed further below. A caveat with these findings, however, is the fact that IL-1 or IL-6 inhibitors have been reported to suppress SARS-CoV-2 neutralizing antibodies in patients with COVID-19 [101]. Hence, the timing of any therapy aimed at suppressing these cytokines will have to be judiciously monitored in order not to interfere with anti-SARS-CoV-2 antibody therapies [98].

### 8.3. CA as a Potential Anti-Infectious Agent against SARS-CoV-2 by Inhibiting Binding to ACE2

**Figure 8:**
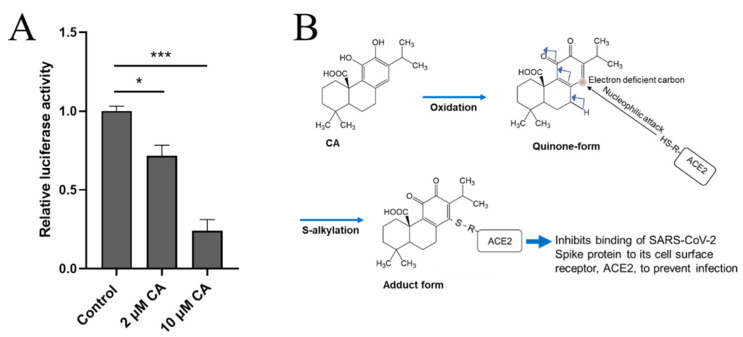
**CA inhibits SARS-CoV-2 infection.** (**A**) Inhibition of SARS-CoV-2 infection by CA in pseudovirus entry assay. HeLa cells stably-expressing ACE2 (HeLa-ACE2) were incubated with SARS-CoV-2 Spike (D614) pseudovirus particles in the presence and absence of the indicated concentration of CA, as described previously [102]. After 48 h, viral transduction efficiency was monitored by luciferase activity. Data are mean ± S.E.M., * *p* < 0.05, *** *p* < 0.001 by ANOVA with Tukey’s multiple comparisons post hoc test; *n* = 3 biological replicates. (**B**) Postulated mechanism of action of CA in preventing Spike protein of SARS-CoV-2 from binding to ACE2. The reaction mechanism involves nucleophilic attack of cysteine thiol of ACE2 on the electron deficient carbon of the quinone formed by oxidation of CA.

Recently, two of us (C-k.O. and S.A.L.) found that CA can inhibit infection with SARS-CoV-2, approaching 90% efficacy, as assessed in a pseudovirus entry assay (Figure 8A). Mechanistically, CA, after conversion to the quinone form, is likely to S-alkylate critical thiol residue(s) on ACE2, the cell surface receptor for SARS-CoV-2 (Figure 8B). This reaction would then block the interaction of ACE2 with the Spike protein of SARS-CoV-2, and thus abrogate viral entry. Further work on reactions of these critical thiol groups in preventing SARS-CoV-2 infection is forthcoming.

## 9. Proposal for Testing Rosemary Extract or CA against SARS-CoV-2 Infection, Long- COVID, and Neurodegenerative Disorders

As we write this review, we are still in the throes of the COVID-19 pandemic with new variants frequently still appearing. While vaccinations and masking have abated the severity of infection in many cases, the unvaccinated are frequently infected and break-through infections of fully vaccinated and boosted individuals can also still occur. Thus, an effective pill to treat COVID-19 infection is still urgently needed. In addition to acute infection, long-COVID, presumably with direct or indirect effects on the brain, is also a persistent and prevalent problem. The neuropsychological symptoms of long-COVID may well stem from innate inflammatory pathways, at least in part attributable to activation of the NLRP3 inflammasome. Moreover, similar inflammatory pathways have been shown to contribute to the pathogenesis of neurodegenerative disorders including AD and PD. Here, we propose that CA from *Rosmarinus officinalis,* an innocuous pro-electrophilic compound that is converted to an active electrophile, can be used for the treatment of acute and chronic COVID-19 manifestations and for chronic neurodegenerative disorders based on the following:

1. CA is a PED and is much safer than other electrophilic NRF2 activators because it is activated only at the site of oxidation and inflammation by ROS, which it then combats [36,37,38,39,40].
2. CA can potently suppress cytokine storm [98,99,100].
3. CA passes through the blood–brain barrier (BBB) with good bioavailability after oral administration [54].
4. CA inhibits activated microglia, in part, via inhibition of the NLRP3 inflammasome [18].
5. Rosemary (containing CA and CS) is recognized as a food additive in the U.S., EU, and Japan [4,5,6].
6. Rosemary (containing CA and CS) is already used clinically as a therapeutic against rheumatoid arthritis in Germany and is on the FDA “generally regarded as safe” (GRAS) list in the U.S. [103,104]. Moreover, CA manifests very few side effects at very high concentrations in two-species toxicity testing.
7. CA and CS have been shown to manifest neuroprotective effects in vivo in multiple models of AD and PD [64,65,66,67,68].

## 10. Conclusions

Carnosic acid (CA), a diterpene found in the herb rosemary, is a safe pro-electrophilic drug (PED) that activates the KEAP1/NRF2 transcriptional pathway, leading to the sustained induction of phase 2 antioxidant and anti-inflammatory enzymes. CA exerts neuroprotective and anti-inflammatory effects and has been proposed to be a potential therapeutic against chronic neurodegenerative disorders, such as AD and PD, and acute and chronic effects of infections such as SARS-CoV-2. Here, we review the evidence and mechanistic considerations supporting the future testing of rosemary extract or CA against these maladies in human clinical trials. The use of rosemary extract in general and CA in particular as an NRF2 activator and anti-inflammatory has gained wide attention due to the virtual absence of side effects and its relatively low economic cost compared to other strategies. The health-promoting effects of CA have been demonstrated in multiple animal models against chronic neurodegenerative disorders. Moreover, inhibition of the NLRP3 inflammasome by CA represents an important molecular target in limiting IL-1β pro-inflammatory cytokine production. This review also highlights the role of the NLRP3 inflammasome in neurological diseases such as AD and PD/LBD, and in the pathogenesis of COVID-19 including so-called ‘long-COVID’ symptoms. CA appears to inhibit the manifestations of SARS-CoV-2 infection by two mechanisms, one at the point of viral entry, possibly through covalent binding to the ACE2 receptor protein, and the other by ameliorating cytokine storm through the inhibition of NLRP3 inflammasome activation. Thus, CA represents a potential therapeutic for COVID-19 as well as for neurodegenerative disorders like AD and PD/LBD.

## Abbreviations

Aβ, amyloid-β; AD, Alzheimer’s disease; ADRS, acute respiratory distress syndrome ; ARE, antioxidant response element; ASC, apoptosis-associated speck-like protein containing a CARD; a-Syn, a-synuclein; CA, carnosic acid; CNS, central nervous system; COVID-19, coronavirus disease 2019; CS, carnosol; DAMP, danger-associated molecular patterns; DMV, double membrane vesicles; EP, electrophile; ER, endoplasmic reticulum; γ-GCS, γ-glutamylcysteine synthetase; GSH, gutathione; GST, glutathione S-transferase; hiMG, human iPSC-derived microglia; HO-1, heme oxygenase; ICAM-1, intercellular adhesion molecule-1; IL-6, interleukin-6; KEAP1, Kelch-like ECH-associated protein 1; LBD, Lewy body dementia; LPS, lipopolysaccharide (endotoxin); MAV, Mitochondrial antiviral-signaling protein; MERS, Middle East Respiratory Syndrome; NF-κB, nuclear factor kappa-light-chain-enhancer of activated B cells; NLRP3, NOD-, LRR- and pyrin domain-containing protein 3; NMDA, *N*-methyl-d-aspartate; NQO-1, NAD(P)H quinone dehydrogenase 1; NRF2, erythroid derived 2-related factor 2; 6-OHDA, 6-hydroxydopamine; PAMP, pathogen-associated molecular patterns; PAT, pathologically-activated therapeutic; PD, Parkinson’s disease; PED, pro-electrophilic drug; PRR, pattern recognition receptor; ROS, reactive oxygen species; SARS, severe acute respiratory syndrome; SARS-CoV-2, severe acute respiratory syndrome coronavirus 2; TLR, Toll-like receptors; TRX, thioredoxin; TNF-*α*, tumor necrosis factor-*α*.
